# Physical Activity and Subjective Vitality in Female University Students: The Mediating Role of Decisional Balance and Enjoyment of the Activity

**DOI:** 10.3390/bs14080685

**Published:** 2024-08-07

**Authors:** Manuel Jesús de la Torre-Cruz, Alba Rusillo-Magdaleno, José Luis Solas-Martínez, José Enrique Moral García

**Affiliations:** 1 Department of Psychology, Faculty of Humanities and Education Sciences, University of Jaen, Las Lagunillas s/n, 23071 Jaén, Spain; majecruz@ujaen.es (M.J.d.l.T.-C.); jsolas@ujaen.es (J.L.S.-M.); jemoral@ujaen.es (J.E.M.G.); 2 Department of Didactics of Musical, Plastic and Corporal Expression, Faculty of Humanities and Education Sciences, University of Jaen, Las Lagunillas s/n, 23071 Jaén, Spain

**Keywords:** stages of change, physical activity, female university students, perceived benefits and barriers, psychological wellbeing

## Abstract

Regular physical activity (PA) improves the psychological well-being of those who practice it. However, female university students are a risk group due to their low level of PA. Based on the transtheoretical model of behavioural change, the main aim of this study was to examine whether the relationship between PA and subjective vitality was mediated by cognitive-emotional variables such as decisional balance (perceived benefits and barriers) and enjoyment associated with PA in a group of female university students. Participants were asked to complete self-administered questionnaires, which were available for one month via a Google Form. The results showed the existence of a statistically significant, relative, and indirect effect between the stage of change and subjective vitality via both mediating variables. Compared to females in the pre-contemplation stage, those in the action and maintenance stages achieved higher subjective vitality scores as a result of the effect of being in a more advanced stage on decisional balance and enjoyment of PA. It is concluded that female university students who reported regular PA found the activity to be more revitalising, stimulating, and exciting; all positive feelings and cognitions that translated into a more energetic and vital perception of themselves.

## 1. Introduction

Regular physical activity (PA) improves the health of those who practice it [1,2,3]. At the physiological level, PA is associated with a reduction in cardiovascular problems, overweight, obesity, hypertension, and type II diabetes. Moreover, PA can reduce some of the most harmful symptoms of cancer [4,5,6]. Regarding the psychological dimension, PA is directly related to perceived quality of life [7], self-esteem [8,9], subjective happiness [10], resilience [11,12], and cognitive function at any age [13,14]. Also, the greater the practice, the less likely the presence of depression-anxiety symptoms [15] and stress [16].

Despite the physiological, psychological, and cognitive benefits, a decline in PA performance is observed throughout adolescence and especially during the transition from adolescence to early adulthood [2,17]. As a result of this decline, the study of PA habits among higher students has become a topic of interest among health and behavioural professionals. Some studies show that the likelihood of being overweight at this age is five times higher than in the general population [18,19]. This negative trend may be detrimental, as PA practice in late adolescence and early adulthood contributes not only to improved physical fitness but also to the acquisition of valuable psychological resources such as self-confidence, behavioural regulation, and perseverance, which are necessary in a period of life characterized by the progressive acquisition of independence and the increasing assumption of personal and social responsibility [17].

University students are a self-identified sedentary group. Only 25% of higher education students meet the criteria to be considered physically active [20], a percentage that could be even lower according to recent PA criteria established by international organizations [21]. Furthermore, female university students are less likely to initiate and perform PA regularly [22]. Radu, Fagaras, and Vandu [23] found that nearly 35% of approximately 500 female students surveyed reported being sedentary, with more than 34% reporting engaging in PA sporadically. Meanwhile, Murphy et al. [24] found that the proportion of females who reported not engaging in recreational PA, both in and out of university, was much higher than their male counterparts. Reasons for this lower participation include lack of time due to academic and domestic responsibilities; the limited number of sports facilities and the travelling distance to them; the financial cost of joining a gym or sports centre; the lack of social support from parents and peers; and simply a lack of motivation or enjoyment in their practice [18,25].

Several conceptual frameworks have been developed to describe and explain how and why people initiate and maintain healthy behaviours. One of these is the transtheoretical model of stages of change [26]. The notion of stages of change represents a temporal dimension that informs a person’s willingness or intention to change their behaviour [27,28]. With regard to PA, the model proposes the existence of five stages of change: pre-contemplation (being inactive and not intending to change; contemplation (being inactive but considering the possibility of starting PA); preparation (being physically active, but intermittently and insufficiently); action (performing PA regularly, although the duration of this behaviour does not exceed six months); and maintenance (PA is integrated as part of the lifestyle and the intention is to maintain its performance) [14,28].

In addition to identifying the stage in which an individual is located, the model takes into account other constructs, such as change processes (cognitive and behavioural strategies), perceived self-efficacy (confidence in one’s own abilities) or decisional balance (consideration of the benefits/pros and barriers/cons associated with performing a particular behaviour), which allude to personal actions and perceptions that predispose one to move from a lower stage to a more advanced one [29,30]. In terms of decisional balance, studies with university students by Horiuchi et al. [20] and Kim et al. [30] have highlighted the presence of higher scores (superiority of benefits over barriers) in the later stages of the model. In line with this, Duan et al. [17] found that young Japanese and German university students reported lower perceived barriers in the maintenance and action stages compared to the preparation, contemplation and contemplation stages, resulting in an increasingly positive decision balance.

In another sense, there seems to be a positive and significant relationship between PA and the enjoyment associated with its performance [31,32,33]. Authors such as Yan et al. [34] and Rodrigues et al. [35] showed that some subjective emotional responses elicited by PA, including enjoyment or fun with its practice, become variables that help to predict its maintenance over time, thus promoting a physically active lifestyle [36]. This enjoyment may even mitigate the decline in frequency and amount of PA associated with increasing age [37]. Experiencing enjoyment or pleasure from PA is important, and even physically active people may reduce or stop PA if they do not find the activity particularly enjoyable [38]. In fact, lack of enjoyment is one of the barriers cited by adults and older adults for doing less or no PA [39].

As previously discussed, PA is directly related to psychological health [40,41,42]. In general terms, psychological well-being is a construct that reports people’s judgements, perceptions or feelings about themselves [43]. Diener et al. [44] have suggested that the repeated practice of PA is a promising way to generate positive affect or emotions, which in turn contribute to increasing certain indicators of well-being [40,43], including subjective vitality. For example, Mavilidi et al. [45] found that students in a group that took active breaks during the school day lasting 8–20 min (e.g., squats and jumping jacks, running on the floor, tabata, etc.) felt much more vital and energetic at the end of these experiences than their peers, who acted as a control group. Similarly, Ekkekakis, Parfitt and Petruzzello [46] reported a significant increase in subjective vitality in students who performed PA at a moderate intensity (75–84% of their maximum heart rate).

More recently, Dodge, Vaylay and Kracke-Bock [47] found that the subjective vitality of a sample of university students was significantly higher at the end of a self-directed PA session than before the session. Chu and Zhang [48] reiterated the existence of a positive relationship between sports club participation, satisfaction with that participation and subjective vitality in a sample of university students, a relationship that was particularly evident for males. In the same direction, and contrary to expectations, Carmignola, Martinek and Hagenauer [49] found that the subjective vitality of a group of university students increased during the period of distance learning caused by the isolation imposed by the COVID-19 pandemic. A detailed examination of the participants’ responses led the authors to conclude that the increased time spent on PA, as a means of compensating for the fatigue caused by distance learning, was the element that contributed to the higher level of vitality.

Previous empirical evidence reports a relationship between PA, cognitive-emotional variables (decisional balance and enjoyment associated with performing PA) and variables related to psychological health, such as subjective vitality. However, we did not identify any studies that focused on analysing the possible mediating role that both decisional balance and enjoyment or pleasure in performing PA may have in the relationship between PA and subjective vitality. Moreover, previous research findings suggest that PA declines with age and certain subgroups such as girls and some ethnic groups (e.g., African, American and Latinos) are less physically active [50]. More recently, Rosselli et al. [51] reported in their study that not only university girls are less physically active, but they also perceive certain barriers to PA as more significant and insurmountable than their male counterparts. In an attempt to fill this gap, the present study had the following aims: (a) To analyse whether the percentage of Spanish female university students classified in the different stages of the transtheoretical model is similar to that observed in previous studies; (b) to examine if there is a positive and direct relationship between PA and subjective vitality and; (c) to investigate whether the relationship between PA and subjective vitality is mediated by cognitive-emotional variables such as decisional balance and enjoyment of PA. With the latter proposal in mind, it was hypothesized that females in later stages of change would enjoy PA more, perceive greater benefits, and encounter fewer barriers related to PA, which in turn would have a positive effect on perceived subjective vitality.

## 2. Materials and Methods

### 2.1. Participants

**Figure 1:**
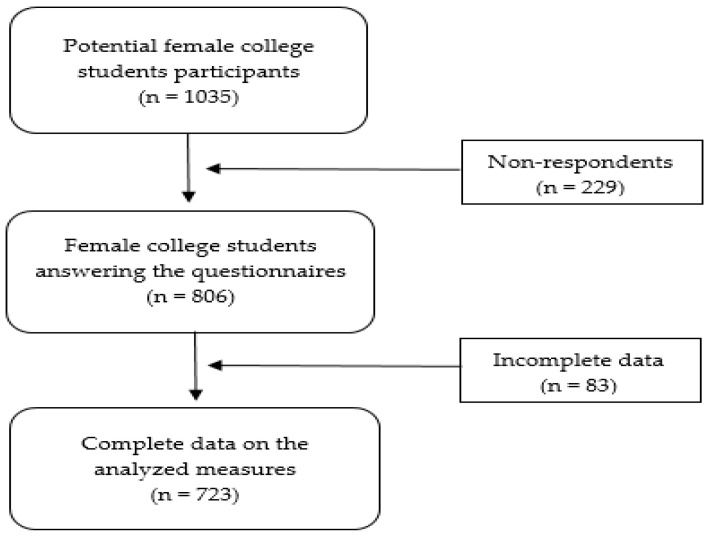
Flowchart of female university students.

Initially, a total of 806 female university students from a single university in the south of Spain participated in this study. The mean age of the participants was 20.16 years (*SD* = 2.08; range = 17–27). All girls were enrolled in the first, second or third year of the following degrees: Bachelor of Psychology (*n* = 254, 30.8%), Bachelor of Early Childhood Education (*n* = 230, 27.8%), Bachelor of Primary Education (*n* = 102, 12.3%), Bachelor of Social Education (*n* = 73, 8.8%) and Bachelor of Social Work (*n* = 64, 7.7%). Participants who did not fully complete some of the measures considered in this study were excluded from further analysis, which reduced the sample size to a total of 723 participants. No other exclusion criteria were considered. Figure 1 shows the flowchart from potential participants to the final sample of university female students.

### 2.2. Procedures

The teaching staff of the different compulsory subjects of the indicated degrees were contacted in order to disseminate and publicize this study among the students. The different measures were made accessible through a Google form “https://forms.gle/4vViRRedkvRfoNbj9 (accessed on 22 March 2022)” entitled ‘Physical activity, self-efficacy and well-being’. For one month (22 March–22 April 2022), the form was available for anyone potentially interested in submitting responses. Participation was voluntary (those who agreed to respond had to give informed consent for the data to be processed) but involved receiving a small ‘extra’ score (0.15 points) to be added to one of the degree subjects. Given the purpose of this particular study, only the responses given by the females were taken into account. The study was carried out in accordance with the Spanish regulations on clinical research involving human subjects (Law 14/2007 of 3 July on Biomedical Research), the legislation on data protection (Organic Law 15/1999) and the principles of the Declaration of Helsinki (Brazil, 2013). It has also been approved by the Bioethics Committee of the University of Jaen, whose reference code is MAY.18/13.PRY (date: May 2018).

### 2.3. Instruments

*Anthropometric information.* Participants provided information about their degree, age, body weight (in kilograms) and height (in metres). The weight and height measurements were used to calculate the body mass index (BMI) according to the Quetelet formula [body weight (kg)/height (m^2^)].

*Stages of Change in Physical Education Questionnaire:* ‘*RM1-FM*’ [52]. Prior to the presentation of the items, participants were shown a definition of PA as a set of activities, such as walking, running, swimming, cycling, etc., in which the effort is at least as intense as those indicated. Initially, participants responded on a dichotomous decision basis (Yes vs. No) to two statements, namely: “I am currently physically active” and “I have tried to be more physically active in the last 6 months”.

In its original format, the test defined regular PA as 30 min or more per day, performed at least five days a week (e.g., one 30-min walk or three daily walks of 10 min each). In light of the new World Health Organization (WHO) recommendations for healthy PA in young adults [21], the previous criteria have been replaced by the following: 30 min of moderate PA (“moderate PA makes you breathe faster but not out of breath. You start to sweat after about 10 min. You can talk but not sing”) every day for 45 min of moderate PA on at least five days per week; or 45 min of vigorous PA on at least three days per week (“vigorous PA makes your breath hard and fast. If you are working at this level, you will not be able to say more than a few words without pausing for breath”).

Based on these modifications, two new questions with a similar response format to the previous one were presented: “Would you say that you currently engage in PA on a regular basis” and “In the past six months, have you engaged in PA on a regular basis”. The combination of answers to each of the four questions made it possible to classify the participant into one of the following stages of change: pre-contemplation (Statement 1 = “no”; Statement 2 = “no”); contemplation (Statement 1 = “no”; Statement 2 = “yes”); preparation (Statement 1 = “yes”; Statement 3 = “no”); action (Statement 1 = “yes”; Statement 3 = “yes”; Statement 4 = “no”); and maintenance (Statement 1 = “yes”; Statement 3 = “yes”; Statement 4 = “yes”).

*Decisional balance*. A Spanish version of the *Exercise Benefits/Barriers Scale* [53,54] was used, with the term “exercise” replaced by “PA”. The Lowell et al. [53] version consists of 43 items: 29 benefits and 14 barriers. Items indicating benefits corresponded to one of the following dimensions: general vital improvement, general improvement in physical fitness, improvement in psychological state, positive social interaction or health prevention. On the other hand, the items indicating barriers were distributed among factors such as the setting in which PA is performed, the time required for its practice, the physical fatigue caused by its performance and the lack of family support. The self-report measure required the participant to indicate their level of agreement (from strongly disagree = 1 to strongly agree = 4) with the proposed statement on a four-point Likert scale. For calculation of the decisional balance score, the “Life enhancement” (“PA allows me to perform daily tasks without feeling tired”) and “Psychological outlook” (“PA improves my mental health”) items of the benefit scales were considered, as well as the items of the obstacle scales “Time expenditure” (“Performing PA takes too much time”) and “Physical exertion” (“Performing PA makes me tired”) items of the obstacle scales. The reliability index values, understood as internal consistency, for each of these scales ranged from 0.83 for “Life enhancement” to 0.75 for “Physical exertion”.

In order to obtain a measure of decisional balance, an average score was first calculated for the two dimensions of benefits and two dimensions of barriers. In both cases, higher scores corresponded to higher perceived benefits and barriers, respectively. For the final calculation of the decisional balance score, the average score for barriers was subtracted from the average score for benefits. A positive score reflected more benefits than barriers (a favourable decisional balance), while the opposite was true for a negative score.

*Enjoyment of physical activity.* A Spanish version of Mullen et al.’s [55] revision of the PACES scale for adults [56] was used. This new instrument consists of a total of eight items headed by the statement “*I find PA…*” and followed by pleasurable adjectives (e.g., stimulating or rewarding) associated with performing PA. The participant was asked to indicate the degree of agreement with each adjective on a seven-point Likert scale (from strongly disagree = 1 to strongly agree = 7). An average score was calculated for the eight items. A higher score corresponded to greater enjoyment of PA practice. The observed reliability index for this study sample was 0.95.

*Subjective Vitality Scale.* The adaptation by Castillo, Tomás and Balaguer [57] of the Subjective Vitality Scale (Individual Difference Level version) developed by Ryan and Frederick [58] was used to assess the subjective experience of feeling vital and full of energy (e.g., “I feel alive and vital”). The instrument required respondents to indicate their level of agreement with the proposed statement on a seven-point Likert scale (from strongly disagree = 1 to strongly agree = 7). Item number two of the scale (“I don’t feel very energetic”), which required reverse coding, had a negative impact on the reliability of the test, so it was deleted before calculating the average score on the scale. A higher score corresponded to higher perceived subjective vitality. Castillo et al. [57] provide information on the validity of the test, given its positive correlation with life satisfaction (*r* = 0.51, *p* < 0.01) and with global self-esteem (*r* = 0.36, *p* < 0.01). The reliability index of the scale in this study was 0.92.

### 2.4. Data Analysis

Descriptive statistics are presented as means and standard deviations for quantitative variables and as frequencies (percentages) for categorical variables. Pearson’s correlation coefficient was used for the initial examination of the relationship between variables. Various analyses of variance (ANOVAs) with post hoc comparisons (Bonferroni correction) were used to examine possible differences in the measures of interest according to the stage of change in which the participant was placed. The values of effect sizes were calculated with *η*^2^ (0.01–0.08, small effect; 0.09–0.24, medium effect; ≥0.25 large effect). Finally, a mediation analysis was conducted to examine, both directly and indirectly, the independent variable (stage of change) on the dependent measure (subjective vitality), with two mediating variables in parallel (decisional balance and enjoyment of PA). Given the categorical nature of the independent variable, the aforementioned mediation analysis was based on the ‘indicator’ coding method (the different levels of the independent variable are compared with one of them, which acts as a referent). All analyses were conducted using SPSS 25.0 for Windows statistical software and the PROCESS macro for SPSS developed by Haynes [57]. The level of statistical significance was set at *p* ≤ 0.05.

## 3. Results

### 3.1. Descriptive Statistics

The mean weight of the participants amounted to 61.05 ± 11.09 kg, while the mean height was 1.63 ± 0.06 m. The formula proposed by Quetelet was used for the calculation of the body mass index (BMI), namely BMI = Mass (kg)/Height^2^ (m^2^). The mean value achieved for this variable was 22.73 ± 3.99 kg/m^2^. The BMI values ranged from a minimum of 15.05 to a maximum of 51.72.

The distribution of females in each of the stages identified by the transtheoretical model was as follows: 14.4% (*n* = 104) were in the pre-contemplation stage, 33.9% (*n* = 245) in the contemplation stage, 13.6% (*n* = 98) in the preparation stage and 5.5% (*n* = 41) were in the action stage. Finally, 32.5% of participants (*n* = 235) were in the maintenance stage.

### 3.2. Correlation Analysis

Pearson’s correlation analysis showed a positive and statistically significant relationship between the variables of age and BMI (*r* = 0.18, *p* < 0.01), with a negative relationship between BMI and enjoyment of PA (*r* = −0.17, *p* < 0.01). Of greater interest, given the purpose of the study, were the relationships observed between measures of enjoyment of PA, decisional balance and subjective vitality. In this regard, positive and statistically significant correlations were obtained between all possible pairs, with *r* = 0.26 (*p* < 0.01) for decisional balance and subjective vitality and *r* = 0.54 (*p* < 0.01) for decisional balance and enjoyment of PA (see Table 1).

### 3.3. Differences in Decisional Balance, Enjoyment of PA and Subjective Vitality as a Function of the Stage of Change in the Transtheoretical Model

Table 2 shows the means, standard deviations (in parentheses), test-retest results, statistical significance, effect size estimates and a-posterior comparisons of decisional balance, enjoyment of PA, and subjective vitality according to the stage of behavioural change. The different univariate analyses of variance showed the existence of statistically significant differences in decisional balance [*F*(1,718) = 25.20, *p* < 0.001, *η*^2^ = 0.12], enjoyment of PA [*F*(1,718) = 20.21, *p* < 0.001, *η*^2^ = 0.10] and subjective vitality [*F*(1,718) = 10.39, *p* < 0.001, *η*^2^ = 0.06].

A post hoc comparison using the Bonferroni correction (0.05/5 = 0.01) revealed a more positive decisional balance for females in the maintenance stage compared to their peers in the pre-contemplation, contemplation and preparation stages. These differences were also statistically significant for females in the preparation and contemplation stages. Also, the enjoyment generated by PA was higher among those who reached the last stage (maintenance) compared to those who were considered inactive (pre-contemplation and contemplation). In addition, those in the preparation and action stages showed greater enjoyment of PA than those in the pre-contemplation stage. Finally, subjective vitality was higher among females who reached the maintenance stage compared to their physically inactive peers (pre-contemplation and contemplation). Similarly, subjective vitality was higher among those in the preparation stage compared to those in the pre-contemplation stage.

### 3.4. Mediation Analysis

The PROCESS macro [59] for SPSS was used to examine both the direct and indirect effects of the independent variable (stage of change) on the dependent variable (subjective vitality) through two mediating variables considered in parallel (decisional balance and enjoyment of PA). As the independent variable was multi-categorical, the “indicator” coding method [60] was used. This procedure juxtaposes the different levels of the independent variable with one that acts as a referent, in this case, the pre-contemplation stage. A 10,000-sample resampling procedure with replacement was used to estimate both the size and statistical significance of the indirect effect.

**Figure 2:**
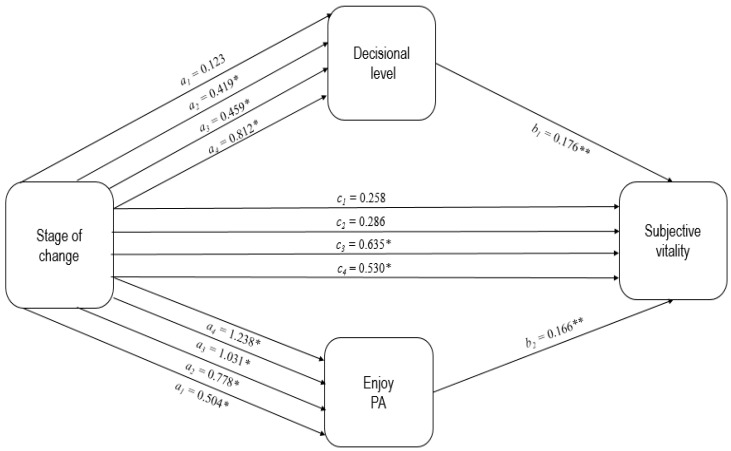
Values of the different coefficients obtained in the mediational analysis (two mediators in parallel). The precontemplation stage was considered as a reference indicator. * *p* < 0.05; ** *p* < 0.01.

The results obtained (see Table 3) showed the existence of a statistically significant indirect relative effect of the stage of change on subjective vitality via both mediating variables. Compared to females in the pre-contemplation stage, those in the preparation, action and maintenance stages had 0.07 [0.013–0.155], 0.08 [0.013–0.178] and 0.14 [0.034–0.259] points higher subjective vitality, respectively, as a result of the effect of being in a more advanced stage on decisional balance, which in turn translated into an increase in perceived subjective vitality. Similarly, participants in the contemplation, preparation, action and maintenance stages scored 0.08 [0.024–0.166], 0.13 [0.048–0.238], 0.17 [0.066–0.306] and 0.21 [0.094–0.348] points higher in subjective vitality due to the positive effect that greater readiness and practice of PA had on the enjoyment associated with PA, which in turn contributed to higher levels of perceived vitality. In addition to the indirect relative effects described above, a direct relative effect of the stage of change on subjective vitality was observed. After adjusting for group differences in decisional balance and enjoyment of PA, those in the action and maintenance stages scored 0.63 [0.168–1.099] and 0.53 [0.218–0.840] points higher on subjective vitality, respectively, than those in the pre-contemplation stage (see Figure 2).

## 4. Discussion

The primary aim of this study was to examine, using the transtheoretical model as a reference, the relationship between belonging to the PA-related stage of behavioural change and subjective vitality in a sample of female university students. Furthermore, it was analysed whether this possible relationship could be mediated by decisional balance (differences between benefits and barriers) as well as enjoyment of PA itself. Preliminary analysis of the results showed that the percentage of female students who reported regular PA (in the action or maintenance stage) was 38%. This percentage is consistent with that found by Kuroda et al. [61] and Horiuchi et al. [20] in their samples of Japanese university students, although it differs from that recently found by Kim et al. [32], in whose research more than 75% of the 414 university students surveyed were in one of the last two stages of the model.

In addition, our analyses showed that 48.3% of the female students attributed a sedentary attitude to themselves (pre-contemplation and contemplation stages). Although this percentage may be considered high, it is similar to that obtained by Duan et al. [17] in their study of a university population. In turn, and despite the fact that almost one in two participants indicated that they did not perform any PA, this result is more favourable than that reported by Guthold et al. [62], whose inactivity rate reached a value of almost 75%.

Although the distribution of participants in the different stages of the model is in line with previous studies, the higher proportion of physically inactive females may be due to the inclusion of more up-to-date and restrictive criteria in this area. This is evidenced by the fact that the statement at the top of the Stages of Change Questionnaire, “30 min of PA at least five days a week”, has been replaced by “30 min of PA seven days a week”, in line with the new WHO recommendations for healthy PA for young adults [21].

In other findings, decisional balance (perception of benefits versus barriers) and enjoyment associated with PA were higher among females for whom PA had become a habit (maintenance stage) compared to those who considered themselves sedentary (pre-contemplation and contemplation stages). A more detailed analysis of the results revealed the existence of an ascending stepwise pattern with regard to decisional balance. Although the scores were always positive, the change was minimal between the pre-contemplation and contemplation stages and between the preparation and action stages. Instead, there were increases between the contemplation and preparation stages and between the action and maintenance stages.

Our findings are consistent with those of Prochaska, Johnson and Lee [63] or Hall and Rossi [64], who reported that perceived benefits increase while costs decrease as one moves to more advanced stages, leading to equivalence between one and the other during the preparation and action stages. Similarly, Duan et al. [17] reported that identified barriers to PA were much lower among university students in the maintenance and exploration (equivalent to action) stages compared to those in the pre-contemplation, contemplation and preparation stages. As noted above, the differences in decisional balance observed in our study occur among participants in the extreme stages as a result of the staggered pattern described. However, in contrast to our findings, Horiuchi et al. [20] found that such differences in benefits and barriers occur among participants in adjacent stages, with lower disadvantages between those in the preparation stage and those in the contemplation stage, and between the latter and those in the pre-contemplation stage.

Similarly, the positive relationship between the stage of change and enjoyment of PA is consistent with the findings of Remmers et al. [65] in primary school children. In their study, they observed a relationship between PA and enjoyment of PA that was moderated by attributed impulsivity, with enjoyment being greater in females who reported greater control over their impulses. More recently, Yan et al. [36] reported that enjoyment of PA mediated the relationship between physical literacy and PA performance in a sample of university students. The authors highlighted the importance of the emotional response associated with PA practice, concluding that even young adults with high levels of physical literacy may not engage in PA if they do not enjoy PA. In contrast to what was observed for decisional balance, the upward trend in the transition between stages for the variable enjoyment of PA was virtually linear, with the greatest increase occurring among members of the two physically inactive stages (pre-contemplation and contemplation stages).

Moreover, females who regularly practised PA, either in a transient temporary period (action stage) or in a long-term period (maintenance stage), expressed a greater sense of subjective vitality than non-practitioners (beyond the possibility of speculating or not on the relevance of starting to practise). This is in line with the results obtained by Molina-García, Castillo and Queralt [66] and Kukic et al. [67] in adolescents and university students. In both cases, they reported a positive relationship between self-reported PA and subjectively attributed vitality. In further support of our findings, the review of studies conducted by Buecker et al. [42] allowed them to conclude the existence of a direct relationship between PA and the positive dimensions of subjective well-being, an association that occurred regardless of the type of PA performed and the participant’s initial level of physical fitness.

In contrast to non-practitioners, females who were used to PA perceived greater functional and psychological benefits and less discomfort from PA. This positive decisional balance in favour of PA in turn led to an additional feeling of subjective vitality. The direct relationship between the stage of change and positive decisional balance has been observed in previous research. For example, the recent systematic review by Sheng et al. [68] and the meta-analysis by Tie et al. [69] confirm the existence of a gradual upward trend in benefits and a downward trend in barriers as one moves to more advanced stages. In this line of argument, and agreement with our findings, Kim et al. [32] found that the benefits of PA were always greater than the barriers and that the benefits were higher in the later stages than in the early stages. Accordingly, the results of our study suggest the presence of a positive decisional balance for each and every stage, although the most significant variations in this dimension occurred between the contemplation and preparation stages, as well as between the action and maintenance stages.

Additionally, the greater the difference between the pros and cons (decisional balance), the higher the subjective vitality that females attributed to themselves. A possible explanation for the existence of this relationship may be due to the nature of the items that make up the two benefit dimensions considered in this study [53]. Both alluded to the possibility that PA could contribute to increased sleep, reduced physical fatigue, improved quality of work, reduced feelings of stress and increased relaxation and mental well-being, all physical and psychological aspects that contribute to a greater sense of energy and vitality.

In parallel, the enjoyment of PA was much higher in females who reached the final stage of the model than in those who were physically inactive and had no intention of changing their behaviour. In addition, this greater enjoyment had a positive effect on the students’ subjective vitality. This finding is consistent with that of Jetzke and Mutz [70]. In their study of young adults, the authors found that those who regularly engaged in PA reported higher indices of psychological well-being, although this relationship was particularly pronounced when practitioners reported doing it for intrinsic reasons, such as distraction or fun. In the same vein, Chu and Zhang [50] reported that greater participation (in terms of quantity and quality) in sports clubs was positively associated with satisfaction with this participation, which in turn was associated with greater subjective vitality, a relationship that was particularly true for male university students. In this line of argument, the work of Lera-López, Ollo-López and Sánchez-Santos [71] highlighted that the association between leisure time PA, subjective happiness and life satisfaction was mediated by perceived health, while Ju [72] concluded that life meaning partially mediated the relationship between PA and subjective vitality in a group of older adults.

In summary, compared to females who did not engage in PA, those who reported regular engagement in PA found the activity to be more revitalising, stimulating, exciting and comforting, all positive feelings or effects that translated into a more energetic and vital perception of themselves.

Considering the PA of female university students is a matter of interest given that girls tend to be physically less active, are less likely to meet the PA criteria recommended by international organizations, and perceive a greater number of barriers to PA (lack of time, lack of support, lack of willpower, and even lack of skill) compared to what is reported by their male peers. Furthermore, this lower amount of PA could be related to a lower degree of psychological well-being. These findings may have implications for the promotion of PA habits among female university students. Such implications include the design of proposals or programmes on the part of the university institution that make PA an enjoyable task or activity, proposals that should also make potential participants aware that its practice has benefits (leisure, social contact) and obstacles (time investment, initial fatigue), although the benefits will increase while the obstacles will decrease as the PA becomes more and more regular.

The study presented here has a number of limitations, the first being the use of a convenience sample (female university students with degrees within the Faculty of Humanities and Educational Sciences of the University of Jaen). Future studies should broaden the range of faculties and degrees and also include males to explore possible gender differences. Nevertheless, the steep decline in PA at this age, particularly among females, conditioned this selection. A further limitation is that the cross-sectional nature of the design prevents the establishment of cause-effect relationships between the variables examined. Future longitudinal studies are suggested in order to clarify causal relationships, although the mediation analysis points to possible directionality. The use of self-report measures may be considered an additional limitation because the information provided by the participant may not reflect the true behaviour due to inaccurate recall or social desirability. In an attempt to minimize this aspect, the procedure ensured the anonymity and confidentiality of participants’ responses. Despite this fact, we believe that these self-report instruments have been found to be valid and reliable in previous research. In addition, the intensity of PA could affect the results. However, measures of intensity of PA were not obtained and only a broad and generic distinction between moderate and vigorous PA was considered. On the other hand, the large sample size and examination of the mediating role of decisional balance and enjoyment of PA in the relationship between PA and subjective vitality are among the strengths of this study. Future studies should examine other correlates (e.g., motivational regulation or PA self-efficacy) between adjacent stages of change for PA practice, as well as analyse potential gender differences. Similarly, it would be of interest to explore the mediating role of perceived family and peer support between stages of change and psychological well-being in the university population.

## Figures and Tables

**Table 1:** Means, standard deviations and Pearson’s correlation coefficients between the different variables.

Correlations							
	Mean	*SD*	Age	BMI	DB	E-PA	SV
Age	20.17	2.08	1				
Body mass index	22.73	3.99	0.18 **	1			
Decisional balance	1.13	0.92	0.05	−0.06	1		
Enjoyment of PA	4.95	1.40	0.01	0.17 **	0.54 **	1	
Subjective vitality	3.95	1.34	−0.05	−0.06	0.26 **	0.28 **	1

BMI = Body mass index; DB = Decisional balance; E-PA = Enjoyment of physical activity; SV = Subjective vitality; PA = physical activity. ** *p* < 0.01.

**Table 2:** Means and standard deviation values (in brackets) for variables decisional balance, enjoyment of PA, and subjective vitality according to the stages of change. Results of ANOVA, *p*-values, and post-hoc comparisons (Bonferroni test).

	PC	CO	PR	AC	MA	*F*(4,718)	*p*	*η* ^2^	Post Hoc
	(*n* = 104)	(*n* = 205)	(*n* = 98)	(*n* = 41)	(*n* = 235)				
Decisional balance	0.74	0.86	116	1.20	1.55	25.19	<0.001	0.12	MA > PC, CO, PR; PR > PC
	(0.88)	(0.94)	(0.84)	(0.83)	(0.78)				
Enjoyment of PA	4.20	4.70	4.98	5.23	5.48	20.22	<0.001	0.10	MA > PC, CO; AC, PR > PC
	(1.36)	(1.43)	(1.22)	(1.28)	(1.26)				
Subjective vitality	3.42	3.78	3.91	4.31	4.31	10.38	<0.001	0.06	MA > PC, CO; AC > PC
	(1.20)	(1.31)	(1.31)	(1.39)	(1.33)				

PC: pre-contemplation; CO: contemplation; PR: preparation; AC: action; MA: maintenance.

**Table 3:** Results of mediational analysis (two mediating variables in parallel) using the ‘indicator’ procedure.

		M1 Decisional Balance				M2 Enjoyment of PA					Y Subjective Vitality		
		Coeff.	*SE*	*p*		Coeff.	*SE*	*p*			Coeff.	*SE*	*p*
Constant	*i*_1_	0.741	0.085	<0.001		4.201	0.131	<0.001			2.594	0.198	<0.001
D1	*a*_1_	0.123	0.101	0.223		0.504	0.156	0.001		c_1_	0.258	0.150	0.087
D2	*a*_2_	0.419	0.122	0.001		0.778	0.187	<0.001		c_2_	0.286	0.181	0.116
D3	*a*_3_	0.459	0.159	0.004		1.031	0.246	<0.001		c_3_	0.635	0.237	0.008
D4	*a*_4_	0.812	0.102	<0.001		1.283	0.157	<0.001		c_4_	0.530	0.158	0.001
M1										b_1_	0.176	0.103	0.005
M2										b_2_	0.166	0.041	<0.001
Constant	*i*_M1_	0.69	0.05	<0.001	*i*_M2_	4.36	0.08	<0.01	*i*_Y_		2.84	0.16	<0.001
		*R*^2^ = 0.123				*R*^2^ = 0.101					*R*^2^ = 0.113		
		*F*(4,718) = 25.20, *p* < 0.001				*F*(4,718) = 20.22, *p* < 0.001					*F*(6,716) = 15.15, *p* < 0.001		

## Data Availability

The original contributions presented in the study are included in the article, further inquiries can be directed to the corresponding author/s.
